# Segmentation of the cervical lesion region in colposcopic images based on deep learning

**DOI:** 10.3389/fonc.2022.952847

**Published:** 2022-08-03

**Authors:** Hui Yu, Yinuo Fan, Huizhan Ma, Haifeng Zhang, Chengcheng Cao, Xuyao Yu, Jinglai Sun, Yuzhen Cao, Yuzhen Liu

**Affiliations:** ^1^ Academy of Medical Engineering and Translational Medicine, Tianjin University, Tianjin, China; ^2^ School of Precision Instrument and Optoelectronics Engineering, Tianjin University, Tianjin, China; ^3^ Obstetrics and Gynecology, Affiliated Hospital of Weifang Medical University, Weifang, China; ^4^ Tianjin Medical University Cancer Institute and Hospital, National Clinical Research Center for Cancer, Tianjin, China

**Keywords:** colposcopic images, cervical lesion, image segmentation, deep learning, feature extraction

## Abstract

**Background:**

Colposcopy is an important method in the diagnosis of cervical lesions. However, experienced colposcopists are lacking at present, and the training cycle is long. Therefore, the artificial intelligence-based colposcopy-assisted examination has great prospects. In this paper, a cervical lesion segmentation model (CLS-Model) was proposed for cervical lesion region segmentation from colposcopic post-acetic-acid images and accurate segmentation results could provide a good foundation for further research on the classification of the lesion and the selection of biopsy site.

**Methods:**

First, the improved Faster Region-convolutional neural network (R-CNN) was used to obtain the cervical region without interference from other tissues or instruments. Afterward, a deep convolutional neural network (CLS-Net) was proposed, which used EfficientNet-B3 to extract the features of the cervical region and used the redesigned atrous spatial pyramid pooling (ASPP) module according to the size of the lesion region and the feature map after subsampling to capture multiscale features. We also used cross-layer feature fusion to achieve fine segmentation of the lesion region. Finally, the segmentation result was mapped to the original image.

**Results:**

Experiments showed that on 5455 LSIL+ (including cervical intraepithelial neoplasia and cervical cancer) colposcopic post-acetic-acid images, the accuracy, specificity, sensitivity, and dice coefficient of the proposed model were 93.04%, 96.00%, 74.78%, and 73.71%, respectively, which were all higher than those of the mainstream segmentation model.

**Conclusion:**

The CLS-Model proposed in this paper has good performance in the segmentation of cervical lesions in colposcopic post-acetic-acid images and can better assist colposcopists in improving the diagnostic level.

## Introduction

According to statistics, cervical cancer is the fourth largest female cancer in the world in terms of morbidity and mortality (1). The WHO currently recommends three different types of screening tests: HPV DNA testing for high-risk HPV types, conventional (Pap) test and liquid-based cytology (LBC), and visual inspection with acetic acid (VIA). The first two methods are complex and expensive to operate. At present, colposcopic directed biopsy is widely used in cervical cancer diagnosis in developing countries. The cases who had a positive test from cytology or HPV test were sent for colposcopy according to ASCCP guidelines (2). At the same time, patients with high risk or uncertainty detected in the first two methods need further examination and treatment under the guidance of colposcopy.

Cervical lesion mainly includes squamous cell cancer and precursors. Referring to the binary classification of the precursors in the 2014 WHO Classification of Tumors of the Female Reproductive System (3), the squamous intraepithelial lesion was histologically divided into the low-grade squamous intraepithelial lesion (LSIL, traditionally called cervical intraepithelial neoplasia (CIN) 1) and high-grade squamous intraepithelial lesion (HSIL, traditionally named CIN 2 and CIN 3). The two-tier system is regarded as more biologically relevant and histologically more reproducible than the three-tier terminology used in the prior edition and is therefore recommended (3). Colposcopic diagnosis requires that the operating colposcopist can accurately determine the characteristics of white epithelial acetate, which largely depends on the clinical experience of colposcopists. In areas with insufficient medical resources, the lack of experienced inspectors and the heavy workload of screening pose great challenges to screening (4). Machine learning algorithms have been proved to be effective in cases where medical diagnosis requires subjective judgment (5). With the development of artificial intelligence technology, computer-aided diagnosis (CAD) based on deep learning has made remarkable progress, which provides a solution to improve the accuracy and stability of diagnosis and reduce the workload of medical personnel. A series of achievements have been made in the computer-assisted diagnosis of colposcopy. However, in the field of deep learning, related studies mainly focused on the gross classification of the lesion based on colposcopic images and the detection of HSIL+ (HSIL and cervical cancer), and there were relatively few studies on the segmentation of the lesion region that could provide intuitive guidance for colposcopists. Accurate segmentation results can provide a good foundation for further research on the classification of the lesion and the selection of biopsy sites. Therefore, cervical lesion region segmentation plays an important role in cervical cancer diagnosis.

In this paper, a deep learning method named cervical lesion segmentation model (CLS-Model) was proposed to segment the cervical lesion region, that is, LSIL+ (including CIN and cervical cancer), in colposcopic post-acetic-acid images to assist colposcopists in accurately locating the lesion region and selecting biopsy sites. It included three parts. First, the improved Faster R-CNN was used to extract the cervical region and remove the interference noises of instruments and vaginal wall in the colposcope pose-acetic-acid images. Second, the cervical lesion segmentation network (CLS-Net) was proposed. EfficientNet-B3 was adopted for the cervical region feature extraction. The features extracted after the 28th layer was fed into the atrous spatial pyramid pooling (ASPP) module to capture multiscale information, and then the features extracted from the 21st layer was added after upsampling to realize cross-layer information fusion. The sample was taken to the size of 640 × 640, to achieve a fine division of the diseased region. Third, the segmentation result was mapped to the original image, which is convenient for doctors to observe. After that, the model was visualized with a heatmap, and the analysis of the HSIL+ recall value proved that the segmentation results of the model could be used to further detect HSIL+ and locate tissue biopsy points.

## Related work

Deep learning has achieved great success in the field of medical image segmentation, and computer-aided diagnosis (CAD) plays an increasingly important auxiliary role in the diagnosis of malignant tumors. In recent years, to diagnose lesions from colposcopic images, researchers have proposed many methods mainly around the cervical region or transformation zone extraction and lesion segmentation.

### Cervical region extraction

Irrelevant information such as the vaginal wall and vaginal dilator in the colposcopic images will disturb the detection of the cervical region. At the same time, the lesion region may be outside the transformation zone. Therefore, extracting the cervical region is very important for the detection of the lesion. Traditional methods are used to segment unlabeled data. For example, Sumindar et al. (6) proposed a method using color features, morphological operations, and Gaussian mixture model (GMM). Mercy et al. (7) used the Gabor filter method. Meanwhile, most researchers have used K-means, which is a machine learning algorithm (8, 9), and (10). However, these methods are sensitive to noise and have the defect of over-segmentation. For labeled data, previous studies mainly used Faster R-CNN (5, 11), to extract the cervical region.

### Lesion region segmentation

The current research is mainly divided into the segmentation of the acetowhite region and LSIL+.

Shi et al. (12) segmented the acetowhite region by combining the features of gray-level symbiosis and the level set algorithm. Yue et al. (13) first generated an attention map based on CICN combined with UNet and CAM blocks and then segmented the acetowhite region through the proposed AWL-CNN network. Liu et al. (10) used DeepLabV3+ to segment the acetowhite region, which included the lesion region and inflammation, partial normal metaplastic squamous epithelium region leucoplakia, and other non-lesion regions. Therefore, segmentation of the acetowhite region alone cannot provide doctors with a more accurate lesion-assisted diagnosis.

At present, the segmentation of cervical precancerous lesions is mainly divided into region-based and pixel-based methods.

Based on region segmentation, in 2011, Sun et al. (14) generated anatomical maps based on color and texture, using K-means means clustering to further cluster the adult region of the tissue region defined by anatomical feature maps, combining adjacent region classification results by probability based on CRF classifiers and determining the final classification results by KNN and LDA integration. In 2021, Roser et al. (15) proposed using PCA to reduce the dimensionality of the RGB vector and used an ANN to generate the probability map of the precancerous lesion for each pixel. Then, seed point region growth was used to connect the points exceeding the threshold value to the segment region, and whether HSIL+ was determined according to the size of the lesion region, but HSIL+ had nothing to do with the size of the region. It is also affected by noise, is prone to cavities and over-segmentation, and has high requirements on the results of ANN extraction.

Based on region segmentation, in 2018, Zhang et al. (16) used cam-based localization of the lesion region, but only general localization of the lesion was carried out without a specific contour. In 2020, Xue et al. (17) adopted UNet, and Zhang et al. (18) proposed an improved UNet by adding two convolution blocks at the input and output based on the original UNet to better extract image feature information. Yuan et al. (19) replaced the encoding part of UNet with ResNet to segment CIN 1+. However, they only fine-tuned UNet and did not attempt to compare and improve it with other segmented networks.

## Methodology

**Figure 1:**
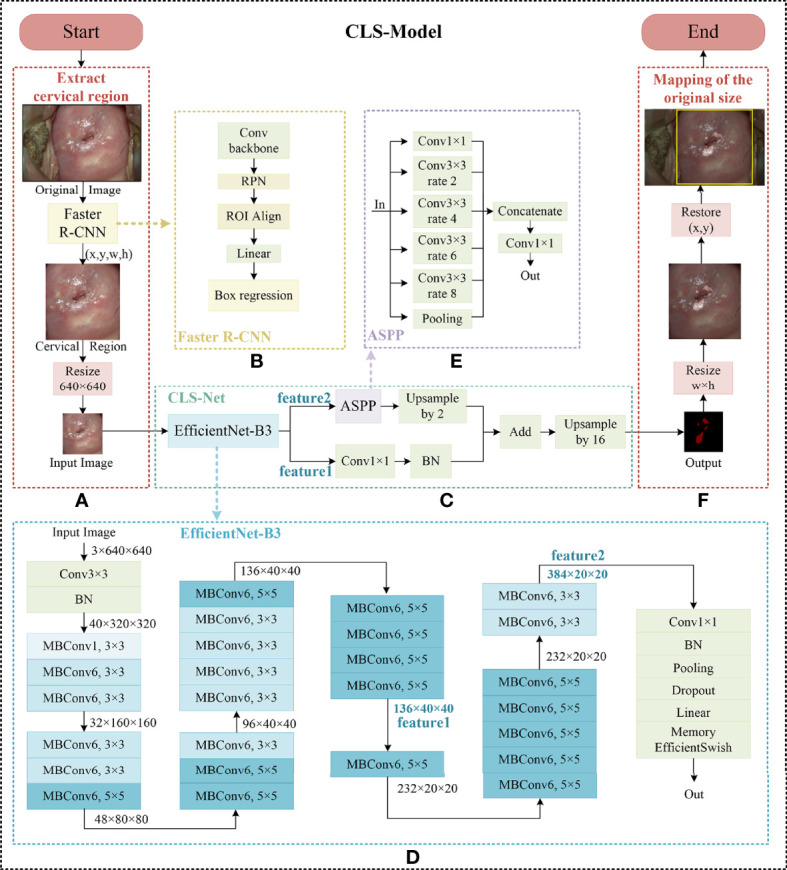
The overall architecture of CLS-Model. **(A)** The architecture of the cervical region extraction model, **(B)** the improved Faster R-CNN, **(C)** CLS-Net, **(D)** EfficientNet-B3, **(E)** ASPP, and **(F)** mapping (the yellow box is the cervical region, and the pink-white region is the lesion. Normal region is indicated by a translucent gray mask).

Our proposed segmentation method consisted of three parts: extraction of the cervical region, segmentation of the cervical lesion network, and mapping of the original image. First, the improved Faster R-CNN was used to extract the cervical region in the images. Second, the cervical lesion segmentation network CLS-Net proposed was used to segment LSIL+. Third, the image was restored and mapped to the original image according to the zoom ratio. The overall architecture of the CLS-Model is shown in **Figure 1**.

### Extraction of the cervical region

The shape of the cervical region in the colposcopic post-acetic-acid image was irregular, and the data set was marked with rectangular boxes by experienced colposcopists to facilitate subsequent processing. Thanks to the data set marked by experienced colposcopists, the supervised learning Faster R-CNN target detection method was used to detect the cervical region. Compared with k-means clustering and other segmentation methods with irregular cervical boundaries (20), the rectangular segmentation results were more convenient for subsequent experiments. Compared with Faster R-CNN and other target detection algorithms, the improved Faster R-CNN method using ROI Align technology referencing Mask R-CNN has higher detection accuracy when the time is similar (21). Since there was only one category of regression box, namely, the cervical region, the branch of classification was deleted in this paper to reduce the complexity and computation of Faster R-CNN on the basis of ensuring accuracy.

In this paper, the improved Faster R-CNN model was used to extract the cervical region. Due to the large size of the original colposcopic post-acetic-acid image and the size of the extraction region, and the scaling did not affect the subsequent analysis of the lesion, the extraction region was uniformly reduced to 640 × 640, which was convenient for further processing of the subsequent segmentation network under the premise of maintaining clarity. The overall framework of the cervical region extraction model is shown in **Figure 1A**, and the structure of the improved Faster R-CNN is shown in **Figure 1B**. The coordinates of the upper left corner point of the rectangular frame and the width and height of the rectangular frame were recorded when the cervical region was extracted, denoted as (x, y, w, h).

### Cervical lesion segmentation network

#### Overall framework of the CLS-Net model

The CLS-Net model used an end-to-end encoder–decoder structure, and the overall framework is shown in **Figure 1C**. In the encoding part, the efficient and accurate EfficientNet-B3 (22) network was used to extract the features of the cervical lesion region. The size of the feature map on the 20th layer was 1/16 of the original size as feature1, and that on the 28th layer was 1/32 of the original size as feature2. The two extracted layers were the two smallest sizes in the process of subsampling and were the last layer under this size, with better deep pragmatic features. Low-level features had higher resolution and contained more location and detailed information. However, due to less convolution, they had lower semantic information and more noise. High-level features had stronger semantic information but lower resolution and poor ability to perceive details. Therefore, a multiscale feature fusion method across layers was used in the decoding part. The size of feature1 was 1/16 of the original image size after the convolutional layer and BN layer with a convolutional kernel of 1 × 1, which was consistent with the 1/16 of the original size obtained by feature2 through the ASPP module designed for this lesion after two upsampling layers. The characteristic information of the adjacent high and low layers was fused, and finally, the sample was upsampled 16 times to the original size. The EfficientNet-B3 module and ASPP module are described in detail below, respectively.

#### EfficientNet-B3 module

EfficientNet (22) is a standardized model extension method that strikes an excellent balance among the three dimensions of model width, depth, and resolution. EfficientNet uses MBConv in MobileNetV2 (23) as the backbone network of the model, and the squeeze and scheduling method in SENet (24) is used to optimize the network structure. The number of parameters in EfficientNet is greatly reduced compared to other models, which greatly improves the operating efficiency of the model and greatly reduces the threshold for model deployment. For the various networks in ImageNet’s history, EfficientNet has been effective in crushing (22).

EfficientNet-B0 is a baseline model developed through AutoML MNAS. In this paper, ImageNet pretrained EfficientNet-B3 was used to realize feature extraction. The model had a total of 34 layers, and only the one before the 28th layer was used in this paper, as shown in **Figure 1D**.

The input image size was 640 × 640, the size of the 20th layer after feature1 was 40 × 40, which was 1/16 of the original image size, and the size of the 28th layer after feature2 was 20 × 20, which was 1/32 of the original image size. These two layers were the last layer under this size, containing the deepest semantic information under this size.

#### ASPP module

Atrous Spatial Pyramid Pooling (ASPP) is a module to sample the input feature graph in parallel with the dilated convolution of different sampling rates, concatenate the obtained results together to expand the number of channels, reduce the number of channels to the number of output channels (class number) through a 1 × 1 convolution, and capture image feature information through multiple scales.

In this paper, based on the characteristics of a large area difference in the lesion region and the size after subsampling, the ASPP module suitable for the sample rate of the lesion was redesigned based on the ASPP proposed by DeepLabV3+, including six branches, a 1 × 1 convolution, four 3 × 3 dilated convolutions at rates = {2,4,6,8}, and one global average pooling. Then, we used a convolution fuse and concatenated the features of six branches to capture multiscale information. After that, we reduced the number of channels to half of the input layer through a 1 × 1 convolution. The architecture of the ASPP module is shown in **Figure 1E**.

The size of the input image was 640 × 640, and the feature size after the 28th layer was 20 × 20. When the subsampling rate was 2, 4, 6, and 8, the size of the equivalent convolution kernel was 5 × 5, 9 × 9, 13 × 13, and 17 × 17, respectively, which was sufficient to fully extract the features of this layer. Therefore, subsampling rates of 2, 4, 6, and 8 were adopted in this paper to capture multiscale information.

### Mapping of the original image

After the segmentation result image with the size of 640 × 640 was obtained, the segmentation image was mapped to the original image according to the coordinates of the upper-left-corner point of the rectangular frame and the width and height of the rectangular frame (x, y, w, h) for the convenience of the colposcopists. The normal region was superimposed with translucent black masks, and the lesion region was not superimposed with any original appearance. As shown in **Figure 1F**.

## Experiments

### Dataset

Data were collected from 12,572 cases of colposcopy provided by the Cervical Disease Center, Affiliated Hospital of Weifang Medical College, China, from July 2013 to May 2021. After screening, 11,510 cases remained, including 4,504 normal cases, 5,338 LSIL cases, and 1,668 HSIL+ cases. Data screening criteria were as follows: patients with necessary information (patient age, HPV test results, cervical cytology results, cervical transformation zone type, colposcopic images, colposcopic pathological results, biopsy pathological report) and qualified colposcopic images (The cervix was clear and intact, no severe bleeding, and the lesion was not severely covered by leucorrhea.) were selected.

**Figure 2:**
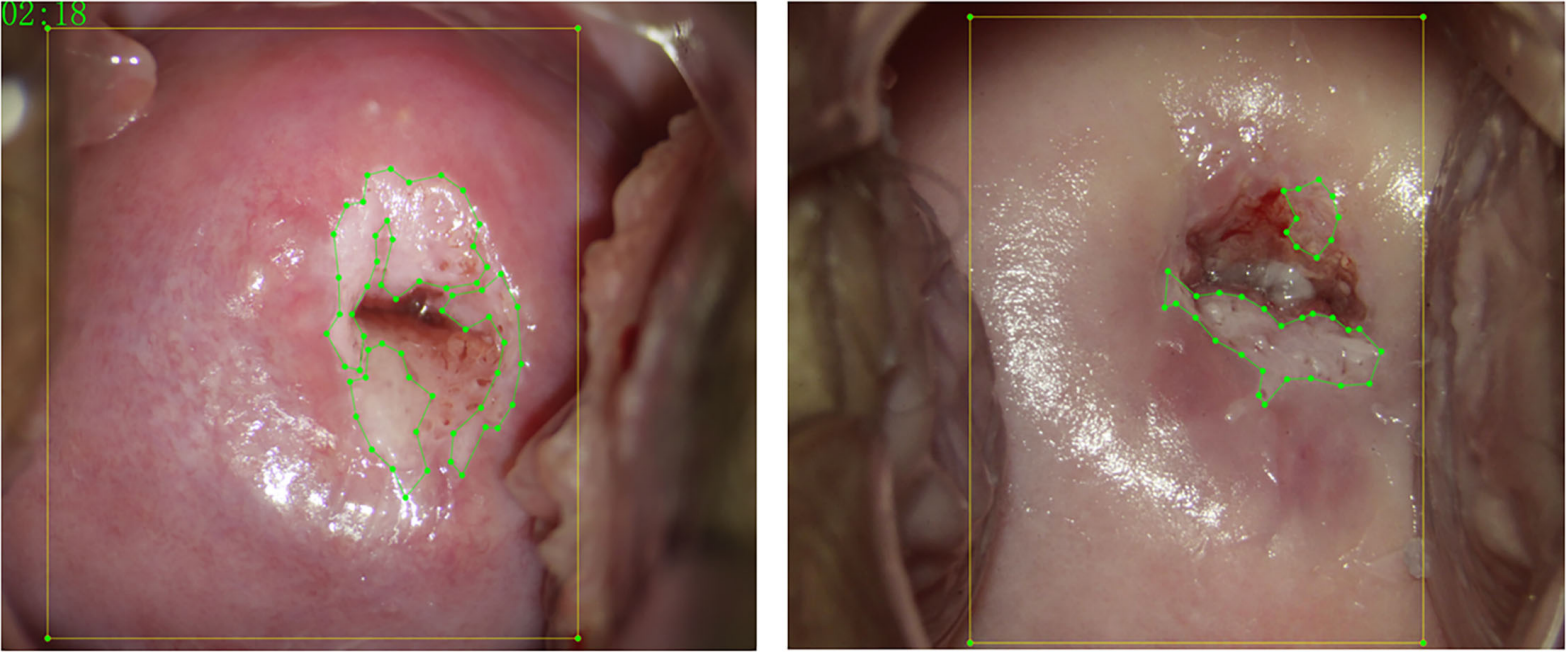
Annotation schematic diagram.

Each case contained a biopsy pathological report, a colposcopy pathological report, a post-acetic-acid image (Apply 3%–5% acetic acid solution for 1 min.), and a JSON file labeled with LabelMe software (https://github.com/wkentaro/labelme) for the lesion region and cervical region. The post-acetic-acid images were all 2,656 × 1,992, and the ratio of length to width was 4:3. Images were screened by five colposcopists with more than 3 years of experience. They took biopsy pathological results as the ground truth and used LabelMe software to label the lesion area for each post-acetic-acid image. The final labels were reviewed by a deputy chief colposcopist with more than 10 years of experience. **Figure 2** shows the schematic diagram of the annotation. The yellow box marks the cervical region, and the green point-lines mark the lesion region.

In the first part, the cervical region was extracted. Due to the relatively simple task, the train sets, validation sets, and test sets contained 700, 100, and 200 cervigrams, respectively, out of 11,510 cases. In the second part, since only the lesion region was segmented, 5,455 LSIL+ cervigrams (LSIL and HSIL+) were selected for the experiment after excluding the small and scattered lesion region, menopause, severe inflammation, and other samples that were difficult to label, and they were divided into 3,820 train sets, 545 validation sets, and 1,090 test sets at a ratio of 7:1:2. To reflect the generalization ability of the model, the train sets and validation sets were shot with Leisegang equipment, and the test sets were shot with OPTOMIC-OP-C5 equipment. **Table 1** summarizes the image distribution. The Ethics Review Committee of Tianjin University granted ethical approval for the study (TJUE-2021-136).

**Table 1:** The image distribution.

Part	Train sets	Validation sets	Test sets	All
Extract cervical region	700	100	200	1000
Segment lesion region	3820	545	1090	5455

### Experimental setup

The network was implemented in Python 3.7, PyTorch v1.7.0 library, torchvision v0.9.0, Matplotlib v3.4.4, NumPy v1.19.2, efficientnet_pytorch, and Cuda v11.0 with an NVIDIA GeForce RTX 3090 graphics card and 24-GB memory. All methods were measured on the same platform. We used adaptive moment estimation (ADAM) as the global optimizer. The initial learning rate was set to 0.001 and attenuated to 0.001 after 20 epochs. The weight decay was set to 0.0001. The input images were resized to 640 × 640. All networks were trained with 50 epochs and a batch size of 16. The loss function of the train set and validation set was Dice Loss.


(1) Dice Loss=1-Dice

### Evaluation criteria

The five commonly used criteria, namely, dice, accuracy, recall, precision, and specificity, were employed to evaluate the performance of different models, the details of which are as follows:


(2) $$\text{Dice}=\frac{2\times TP}{FP+FN+2\times TP}$$

(3) $$\text{Accuracy}=\frac{TP+TN}{TP+FP+TN+FN}$$

(4) $$\text{Recall}=\frac{TP}{TP+FN}$$

(5) $$\text{Precision}=\frac{TP}{TP+FP}$$

(6) $$\text{Specificity}=\frac{TN}{TN+FP}$$

(7) $$\text{Score}=\frac{\text{Dice}+\text{Recall}}{2}$$

where TP, TN, FP, and FN are true positive, true negative, false positive, and false negative, respectively. The lesion region is positive and the normal region is negative. Dice is very important in the segmentation process, and cervical precancerous lesions require a low rate of missed diagnosis but a misdiagnosis rate in a reasonable range. Thus, the Recall must be high, and the Specificity just needs to be in a reasonable range. Therefore, we selected the network model with the highest score on the validation set to use on the test set.

### Results and analysis

**Figure 3:**
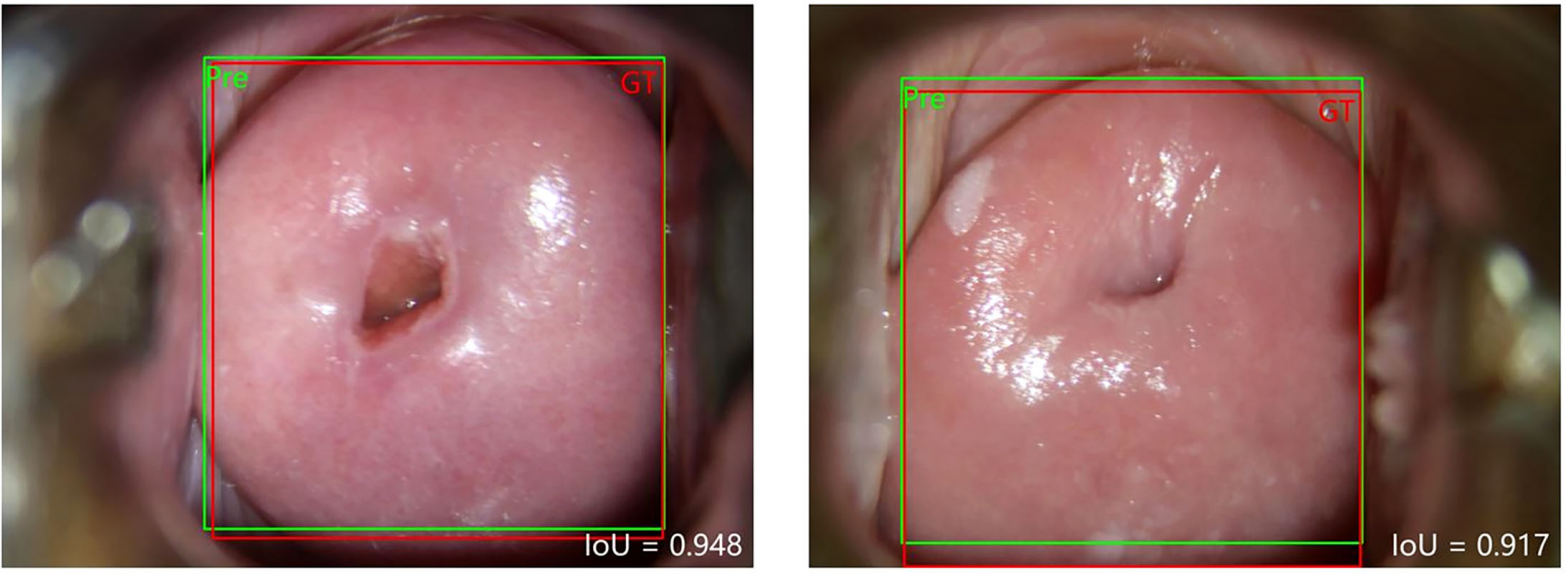
The graphical result of the improved Faster R-CNN.

First, we used the improved Faster R-CNN to extract the features of the cervical region, which obtained the AP@0.8 = 0.995. This means that only one of the 200 test sets has an IOU less than 0.8. The AP@0.8 = 0.995, which is sufficient to satisfy our requirements. The ground truth box (red GT) and prediction box (green Pre) are shown in **Figure 3**. The AP@0.8 = 0.98 in the original Faster R-CNN. It can be seen that the average precision (AP) is improved by using ROI Align for correction.

**Figure 4:**
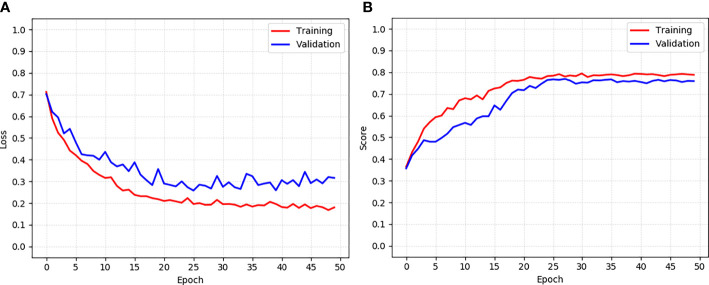
The training and validation loss and score curve of the CLS-Net. **(A)** Loss curve. **(B)** Score curve.

Second, the training and validation loss and score curve of the proposed model CLS-Net are shown in **Figure 4**. The loss function curves of the training set and verification set tend to converge at the 25th round. The calculation methods of loss and score are shown in (1) and (7). The training set reached the maximum value of 0.794 in the 30th epoch, and validation reached the maximum value of 0.769 in the 27th epoch. Therefore, the 30th epoch model with the largest score in the validation set was selected as the optimal model and tested in the test set with a result of 0.764.

**Figure 5:**
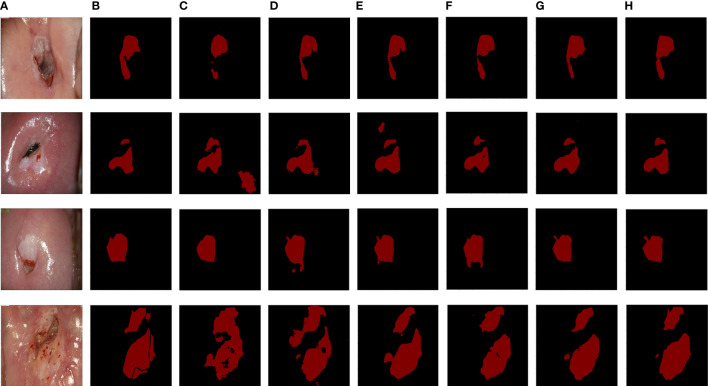
The segmentation results from six methods. **(A)** Original image. **(B)** Ground truth. **(C)** UNet. **(D)** FCN8x. **(E)** DeepLabV3+. **(F)** SegNet. **(G)** CCNet. **(H)** CLS-Net.

The segmentation performance of the proposed CLS-Net was compared with the state-of-the-art segmentation methods, such as UNet (25), FCN8x (26), DeepLabV3+ (27), SegNet (28), and CCNet (29). The performance according to the Dice, Recall, Specificity, and other metrics is shown in **Table 2**. The mean ± std was used to summarize the results. Several segmentation results are presented in **Figure 5**.

**Table 2:** The metrics of CLS-Net and the state-of-art methods in our dataset.

Method	Accuracy	Precision	Recall	Specificity	Dice
UNet (25)	0.9073	0.6941 ± 0.2321	0.6593 ± 0.2233	0.9575 ± 0.0223	0.6307 ± 0.2175
FCN8x (26)	0.9094	0.7102 ± 0.2287	0.6434 ± 0.2097	0.9522 ± 0.0185	0.6311 ± 0.2059
DeepLabV3+ (27)	0.9083	0.6889 ± 0.2101	0.6828 ± 0.1945	0.9545 ± 0.0167	0.6416 ± 0.1816
SegNet (28)	0.9097	0.6867 ± 0.1898	0.7057 ± 0.1733	0.9517 ± 0.0117	0.6600 ± 0.1637
CCNet (29)	0.9191	0.7264 ± 2.003	0.7179 ± 0.1898	0.9560 ± 0.0196	0.6849 ± 0.1802
**CLS-Net (ours)**	**0.9304**	**0.7478 ± 0.1551**	**0.7802 ± 0.1526**	**0.9609 ± 0.0120**	**0.7371 ± 0.1486**

The best performing in each column (evaluation index) are in bold.

**Table 2** shows that the values of the five metrics of CLS-Net are higher than those of the other five models. In the segmentation field, the Accuracy and the Dice coefficient are important indicators to evaluate segmentation. The larger the Accuracy and Dice are, the better they are. In clinical practice, the higher the Recall rate is, the better it is, whereas the Specificity is in the appropriate range. Doctors worry more about missed diagnoses than misdiagnoses. Thus, the model CLS-Net we proposed has the highest Dice of 0.7371, which is 0.0522 superior to the second-best model CCNet and 0.1064 superior to the worst model UNet. It shows that the gap between the CLS-Net’s prediction and ground truth is minimal. However, we found that compared with the segmentation tasks in other fields, the Dice of colposcopic images in all models was generally low, which may be due to the unclear lesion contour and interferences such as inflammation, reflection, or bleeding. CLS-Net has the highest Recall of 0.7802, which is 0.0623 higher than the second-best model CCNet and 0.1368 higher than the worst model FCN8x. This means that CLS-Net has the lowest missed diagnosis rate. In terms of Specificity, all models performed well, with values higher than 0.95, which met the appropriate range with little difference. This proves that the misdiagnosis rate of all models is very low. Finally, the Precision of CLS-Net is 0.7478, 0.0214 higher than that of the second-best model CCNet and 0.0611 higher than that of the worst model SegNet. This indicates that CLS-Net has the lowest false positive rate; in other words, the positive prediction of model CLS-Net is more reliable and can avoid overtreatment of patients.

A finer profile can help doctors make more accurate diagnoses while also making them more difficult to segment. The data set used in this study was labeled as a fine outline of the lesion region after acetic acid was applied under colposcopy, distinguishing between the normal metaplastic squamous epithelium and lesion region that also occur with acetate whiteness, in the hope of giving doctors more accurate auxiliary diagnostic information. As shown in **Figure 5**, the visible partial model segmentation results contain a scaly normal metaplastic squamous epithelium region that is very similar to the lesion region, resulting in decreased accuracy of the segmentation results. The CLS–Net proposed in this paper has made a good distinction between the lesion region and the normal metaplastic squamous epithelium region, and its segmentation results are most consistent with the ground truth in the comparative segmentation model. UNet (25) integrates more low-level features, which is suitable for the target of a relatively stable internal structure of the human body and is not satisfactory for cervical lesion regions of different shapes and sizes in this study.

### Ablation experiments

To verify the effectiveness of our proposed method, we performed ablation experiments on cervical region extraction and ASPP respectively. The results are shown in **Table 3**. Only using the improved Faster R-CNN to extract the cervical region and then segmentation can improve the accuracy by 0.79% and Dice coefficient by 0.0070. The accuracy and Dice coefficient can be improved by 1.14% and 0.0096, respectively, by using ASSP alone. It can be seen that it is effective to use the improved Faster R-CNN and ASPP to optimize the model, but the excellence of the model mainly comes from the design of the overall network structure.

**Table 3:** Ablation experiments on the improved Faster R-CNN and ASPP.

CLS-Model	Accuracy	Precision	Recall	Specificity	Dice
Without Faster R-CNN and ASPP	0.9162	0.7241 ± 0.2337	0.7553 ± 0.1818	0.9562 ± 0.0201	0.7195 ± 0.1875
Without Faster R-CNN	0.9276	0.7392 ± 0.1827	0.7660 ± 0.1978	0.9582 ± 0.0145	0.7291 ± 0.1773
Without ASPP	0.9241	0.7331 ± 0.1912	0.7633 ± 0.1844	0.9578 ± 0.0172	0.7265 ± 0.1716
**All (ours)**	**0.9304**	**0.7478 ± 0.1551**	**0.7802 ± 0.1526**	**0.9609 ± 0.0120**	**0.7371 ± 0.1486**

The best performing in each column (evaluation index) are in bold.

### Feature visualization

**Figure 6:**
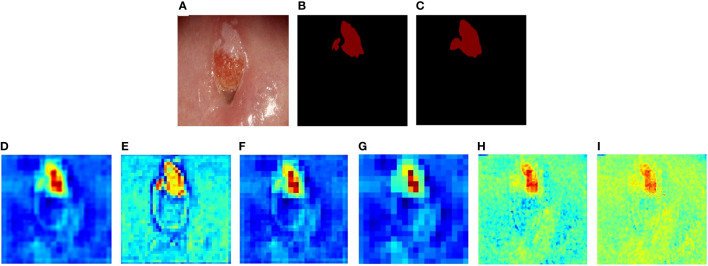
The heatmaps of CLS-Net’s features. **(A)** Colposcopic post-acetic-acid images. **(B)** The ground truth. **(C)** The result of CLS-Net. **(D)** The heatmap of feature2 unsampled by 2. **(E)** The heatmap of feature1 after going through the convolution layer and BN layer. **(F)** The result of feature1 adds up to feature2. **(G)** The heatmap after upsampling to the size of the original image. **(H)** The heatmap of the 8th layer output. **(I)** The result of **(F)** adds up to **(H)**.

We visualized the features extracted from CLS-Net by generating heatmaps. The redder the region is, the greater the contribution of the region to the final classification of the model, and the bluer the region is, the lesser the contribution of the region to the final classification. That is, the model will be judged more by the red area. As seen in **Figure 6**. **Figures 6A-C** are the post-acetic-acid image, the ground truth, and the prediction segmentation, respectively. **Figure 6D** is the heatmap of feature2 unsampled by 2, and **Figure 6E** is the heatmap of feature1 after going through the convolution layer and BN layer in **Figure 1C**. They add up to **Figure 6F**, which combines the deeper semantic features of (d) with the more detailed features of (e) and has better segmentation results. **Figure 6G** is the heatmap after upsampling to the size of the original image. It takes the bilinear difference method and looks smoother.

We also produced other CLS-Net layer features using heatmaps of cross-layer connections in **Figures 6H, I**. **Figure 6H** shows the 8th layer of EfficientNet-B3 which has a size of 80 × 80 and connects feature 1, which also has a size of 80 × 80 and is unsampled by 2. The same connection method is used. As can be seen from **Figure 6H**, the effect is not as good as before. The size of 160 × 160 has worse performance than 80 × 80, as shown in **Figure 6I**. Thus, we only use the layer that has a size of 40 × 40 for cross-layer connections.

## Discussion

### Recall rate is generally low

Although the proposed model shows better segmentation than other comparison models, its Recall rate is still low and its Specificity is high. We found that the model in the prediction was easy to identify some LSIL that could not be distinguished from the normal metaplastic squamous epithelium as normal, lost part of the edge of LSIL, or lost LSIL with a very small region and shallow color and texture features. In addition to the improvement of the model, there may be two reasons. Doctors need to combine iodine images and post-acetic-acid images in high-definition resolution to compare repeatedly to further distinguish normal, LSIL, and HSIL+ in the uncertain region.

It is indeed impossible to make a particularly accurate judgment only from post-acetic-acid images. Second, doctors cannot accurately remove some normal regions that may exist inside the lesion region when labeling, but the neural network model can, which leads to more accurate segmentation of the neural network model but with a lower Recall. However, in clinical practice, there is no requirement for the detection rate of LSIL, and only the detection rate of HSIL is required to be greater than 65% (30), which means that as long as the real region of HSIL+ has a high Recall rate in the lesion segmented by the model, it is ok. We divided 1,090 test sets between LSIL and HSIL+, of which 938 were LSIL and 152 were HSIL+. The formula of HSIL+ Recall rate (HR) is as follows:


(8) $$\text{HR}=\frac{{\text{A}}_{\text{H}}\cap {\text{A}}_{\text{a}}}{{\text{A}}_{\text{H}}}$$

where AH is the real region marked by HSIL+, and Aa is the region of the lesion predicted by the model. The HR values of 152 cases of HSIL+ were statistically analyzed, as shown in **Table 4**.

**Table 4:** HR in 152 cases of test HSIL+.

HR values	Number	Percentage
(0.9, 1.0]	146	96.05%
(0.8-0.9]	2	1.32%
(0.7-0.8]	0	0.00%
(0.6-0.7]	3	1.97%
(0.0-0.6]	1	0.66%

Among the above, 96.05% were greater than 0.9; 0.8 to 0.9 accounted for 1.32%, due to the boundary between some HSIL+ and normal metaplastic squamous epithelium and the columnar epithelium was not easily distinguished, or there was blood, which was predicted to be normal by the model; 0.7 to 0.8 accounted for 0.00%; and 0.7 or fewer accounted for 2.63%. The reason was that the lesion was blocked by a large amount of bleeding, and the bleeding region was labeled HSIL+ when doctors labeled it. However, the bleeding region was not identified as a lesion in the prediction of the model, or it was difficult to distinguish the bleeding region from the normal metaplastic squamous epithelium at the cervical mouth, resulting in a low Recall. In conclusion, although the Recall of the model is not high, the percentage of HR >0.9 is very high, which means that the HSIL+ region can be segmented by the model, and most of the missed region is the LSIL region. Further HSIL+ detection and biopsy site location and other related studies can be effectively carried out on segmented lesions to meet clinical needs.

### Comparison of the proposed model with recent methods

We compared some recently published papers. Since their code is not open source and their datasets are different, we have only listed the results presented in the original literature in **Table 5**.

**Table 5:** Comparison of the proposed model with recent methods.

Year, author	Accuracy (%)	Object of segmentation
2020, Xue et al. (17)	–	Lesion region
2020, Zhang et al. (18)	67.00	Lesion region
2020, Yuan et al. (19)	95.59	Lesion region
2021, Liu et al. (10)	90.36	Acetowhite region
**CLS-Net(ours)**	**93.04**	**Lesion region**

The best performing in each column (evaluation index) are in bold.

In 2020, Xue et al. (17) adopted UNet to segment lesion region subsequent lesion classification, so they only gave the classification results and they did not give the segmentation results on their dataset. Zhang et al. (18) proposed an improved FCN by adding two convolution blocks at the input and output based on the original UNet to better extract image feature information. On their dataset, the accuracy of FCN was 67.00% and UNet was 57.3%. The accuracy of UNet is lower than FCN, which was consistent with the conclusion and analysis of our dataset. Yuan et al. (19) replaced the coding part of UNet with ResNet to segment CIN 1+ whose accuracy could reach 95.59% on their dataset. However, they only fine-tuned UNet and did not attempt to compare and improve it with other segmented networks. In 2021, Liu et al. (10) used DeepLabV3+ to divide the acetowhite area, which is easier than the lesion region, with an accuracy of 90.36%. The accuracy of our method CLS-Net was 93.04%. It can be seen from this that the results of the same method on different data sets differ greatly, so a direct comparison cannot be made. The results of FCN, UNet, and DeepLabV3+ in **Table 2** were the results of emulating the methods they used with our dataset. Their accuracy was 90.94%, 90.73%, and 90.83%, respectively. It can be seen that the proposed method CLS-Net has certain advantages.

### Specular reflection

The specular reflection region usually has high brightness and low color saturation (31), which is easily confused with the coarse white region. Predecessors have also done much research work to remove specular reflection (32). However, we find that almost all models, especially CLS-Net, performed well without removing specular reflections, as shown in **Figure 5**. This indicates that almost all models can effectively distinguish the features of the specular reflection region from the lesion region. This is likely because we have enough finely annotated datasets to make the model have a good ability to learn the different features of the specular reflection region and lesion region. Therefore, it is no longer used as a preprocessing to remove specular reflection in this paper.

### Fine contour labeling

Distinguishing between normal metaplastic squamous epithelium and squamous intracutaneous lesion (cervical lesion) that occur in acetowhite reactions is a challenge for less experienced colposcopists. In other related studies (7), and (12) to (10), the acetowhite region (AW) is generally used as the segmentation target. In this study, normal metaplastic squamous epithelium and squamous intracutaneous lesion region were distinguished, and the segmentation target was the cervical lesion region. It aims to provide doctors with more precise diagnostic information, but it also brings higher segmentation difficulty, resulting in a better segmentation effect of the model for the acetowhite region in this study. In this paper, the CLS-Net model was proposed to pay more attention to the fine features of the cervical lesion region and ignore the interference of the region similar to the lesion, as shown in **Figures 6D–I**, which achieved a better segmentation effect of the actual lesion region and could be used to assist the accurate diagnosis of colposcopists and the teaching and training of colposcopists. It also provides a good foundation for the subsequent research on lesion classification and so on.

## Conclusions and future work

In this work, we proposed a new segmentation method CLS-Model for cervical lesions, which contained three key steps: The improved Faster R-CNN was used to extract the cervical region from colposcopic post-acetic-acid images, which effectively avoided the interference of other tissue equipment on subsequent processing. Based on this, a new segmentation model CLS-Net was proposed, which could effectively segment the lesion region (LSIL+). Finally, the segmentation results were mapped to the original image size. This method had better performance than other similar methods. Unlike other related studies, our solution does not require separate removal of specular reflections, but it does not affect the performance of the model. Our segmentation scheme distinguishes cervical lesion and normal metaplastic squamous epithelium and other atypical tissues and has more refined results than the segmentation of the acetowhite region in other studies. Heatmaps were used to achieve visual interpretation of the model. At the same time, we explored the HSIL+ Recall (HR) which was more clinically valuable using CLS-Net, and it could achieve satisfactory results.

Of course, there are some limitations to our research. Since there is no publicly available dataset with segmentation labels, our model only performs well on our dataset and can only perform simulated experimental comparison according to the method proposed in reference. In the face of more complex data input, the stability of the model needs to be evaluated. At the same time, the fusion model with the hospital information system needs further research in the future. In addition, our current research is only aimed at the automatic segmentation of cervical lesions. In clinical practice, other requirements, such as cervical transformation zone classification and tissue biopsy point recommendation, need to be further studied. We hope that fully automatic cervical detection can be achieved in the future based on the segmentation results.

## Data availability statement

This data set is for the hospital and school research collaboration and is not publicly available at present. Requests to access the datasets should be directed to Yinuo Fan, xiaonuomi@tju.edu.cn.

## Ethics statement

The Ethics Review Committee of Tianjin University granted ethical approval for the study (TJUE-2021-136). Written informed consent to participate in this study was provided by the participants’ legal guardian/next of kin.

## Author contributions

YF: She completed the basic writing, experiment and programming of the paper. HY: He made further revisions and checks on the paper and provided guidance for the writing of the thesis HM: He assisted to complete the algorithm part of innovation and part of the experiment. HZ: She helped annotate the images. CC: She helped annotate the images. XY: He offered medical help. JS: He helped to check the paper. YC: She made further revisions and checks on the paper and contacted the hospital to cooperate. YL: She made further revisions and checks on the paper, helped annotate the images and instructed in medicine. All authors contributed to the article and approved the submitted version.

## Funding

This work was supported in part by Major Science and Technology Projects of Tianjin, China, under Grant No.18ZXZNSY00240, and Science and Technology Projects of Tianjin Health Commission, China under Grant No. TJWJ2021QN009.

## Acknowledgments

We would like to thank all the colposcopists who participated in data screening, labeling, and patient guidance in the cervical Disease Center of Affiliated Hospital of Weifang Medical College.

## Conflict of interest

The authors declare that the research was conducted in the absence of any commercial or financial relationships that could be construed as a potential conflict of interest.

## Publisher’s note

All claims expressed in this article are solely those of the authors and do not necessarily represent those of their affiliated organizations, or those of the publisher, the editors and the reviewers. Any product that may be evaluated in this article, or claim that may be made by its manufacturer, is not guaranteed or endorsed by the publisher.
